# An Empirical Study of Dataset Size Effects on Fine-Tuning Depth in Transfer Learning for Chest X-ray Classification

**DOI:** 10.7759/cureus.106159

**Published:** 2026-03-30

**Authors:** Ackley Dias Will, Jordan Sarkodie

**Affiliations:** 1 Department of Computing, Andrews University, Berrien Springs, USA

**Keywords:** chest x-ray classification, fine-tuning depth, resnet-50, training set size, transfer learning

## Abstract

Transfer learning is a standard approach in medical imaging to address the limited availability of annotated data. However, practical decisions regarding fine-tuning depth are often made heuristically, without clear empirical guidance on how these choices depend on dataset size. This study presents a controlled empirical analysis of the interaction between dataset size and fine-tuning depth in a chest X-ray image classification task using a total of 5856 chest X-ray images. Using a fixed ResNet-50 backbone, we compared linear probing, shallow fine-tuning of the last convolutional block, and deeper fine-tuning of the last two convolutional blocks across multiple data regimes. Linear probing remains competitive in low-data settings. As dataset size increases, performance improves across all strategies, and fine-tuning provides small but consistent gains in ranking-based metrics, including the area under the receiver operating characteristic curve (ROC-AUC) and average precision (AP), relative to linear probing. The gap between shallow and deeper fine-tuning narrows in larger regimes, indicating diminishing returns from additional unfreezing under this experimental setup. Rather than proposing a new model, this work provides practical guidance for selecting fine-tuning strategies based on data availability and system constraints, supporting informed engineering decisions in medical imaging pipelines where trade-offs between performance, complexity, and computational cost must be balanced.

## Introduction

Deep learning based on convolutional neural networks is a central component of modern medical imaging systems [1,2]. In chest X-ray analysis, transfer learning from models pretrained on large-scale natural image datasets (e.g., ImageNet) is widely used to mitigate limited labeled medical data and can provide strong baselines after task-specific adaptation [3,4]. Related benchmarks and large chest radiograph resources, including CheXpert, CheXNet, ChestX-ray8/14, and MIMIC-CXR, illustrate the established use of deep learning in chest X-ray research [5-8].

Despite broad adoption, fine-tuning decisions remain largely heuristic, particularly regarding how many layers of a pretrained network should be unfrozen. Linear probing and partial fine-tuning are both common, but there is limited practical guidance on how the benefit of deeper fine-tuning depends on dataset size. This question is especially relevant in real-world settings where compute budgets, annotation costs, and retraining frequency matter.

Prior work comparing transfer-learning strategies in medical imaging suggests that the benefits of fine-tuning can depend on the task and data regime, motivating controlled analyses across dataset sizes [9]. Motivated by this, we conducted a controlled study that isolates the interaction between dataset size and fine-tuning depth using a fixed architecture and a consistent training pipeline.

More specifically, the objective of this study is to evaluate how the relative benefit of different adaptation depths changes as a function of dataset size in chest X-ray classification. To do so, we compare three practical strategies within the same ResNet-50-based pipeline: linear probing, shallow fine-tuning of the last convolutional block (FT conv5), and deeper fine-tuning of the last two convolutional blocks (FT conv4-5).

This study contributes a controlled empirical comparison of these three transfer-learning strategies across multiple dataset-size regimes in chest X-ray classification. It also provides practical guidance for selecting fine-tuning depth as a function of data availability and system constraints, emphasizing trade-offs in performance, complexity, and diminishing returns.

## Materials and methods

Dataset

Experiments use the chest X-ray subset of the publicly available pediatric imaging dataset released by Kermany et al. [10]. Only chest X-ray images from the dataset's predefined chest X-ray directories were included in the present study, whereas non-chest X-ray images from the broader source release were excluded. Labels included 'NORMAL' and pneumonia subtypes, and bacterial and viral pneumonia cases were merged into a single 'PNEUMONIA' class to form a binary classification task. No additional manual filtering was performed.

The dataset provides a predefined train/test directory split [10]. We used the original training directory and created an internal 80/20 split with a fixed random seed to obtain training and validation sets. The predefined test directory was kept fully held out and remained fixed across all experiments.

Experimental design: data regimes

To analyze data-availability effects, progressively smaller data regimes were constructed from the same training and validation pipelines via batch-level truncation using TensorFlow (tf) data pipelines through tf.data.Dataset.take(). Because truncation is performed over batches, image counts per regime are reported as batches multiplied by batch size, with minor variability possible in the final batch.

All regimes used the same preprocessing, augmentation, architecture, optimization logic, and evaluation protocol; only the amount of training and validation data available to the model differed by regime. Model selection procedures, including early stopping and checkpointing, were driven only by validation performance computed on the corresponding reduced validation subset for each regime. Six data regimes were evaluated with training sizes of 128, 256, 512, 1024, and 2080 images, along with the full regime using 4186 training images, while keeping the held-out test set fixed. The batch size was fixed at 32 for all experiments.

Model architecture

All experiments use ResNet-50 pretrained on ImageNet as the backbone, and input images are resized to 224×224 and preprocessed using the standard ResNet-50 preprocessing consistent with ImageNet training [11,12]. A fixed classification head is attached, consisting of global average pooling, a dropout layer with a rate of 0.25, and a sigmoid output neuron for binary classification. No architectural modifications were introduced across experiments.

Fine-tuning strategies

Three fine-tuning strategies were evaluated. In linear probing (LP), the pretrained backbone is fully frozen, and only the classification head is trained. In shallow fine-tuning (FT conv5), the last convolutional block (conv5) and the classification head are trained jointly. In deeper fine-tuning (FT conv4-5), the last two convolutional blocks (conv4 and conv5) and the classification head are optimized end-to-end, while earlier convolutional blocks remain frozen.

Although additional, deeper unfreezing configurations were explored in auxiliary runs, the main analysis focuses on linear probing, FT conv5, and FT conv4-5 to isolate the primary trade-off between adaptation depth and data availability.

Training configuration

Optimization used the Adam optimizer [13]. For linear probing, the classification head was trained with a learning rate of 1×10⁻³ for up to eight epochs. For fine-tuning, smaller learning rates were used to preserve pretrained representations, with FT conv5 using a learning rate of 1×10⁻⁴ for up to twelve epochs and FT conv4-5 using 5×10⁻⁵ for up to fifteen epochs.

Early stopping, learning-rate reduction, and checkpointing were based on validation precision-recall area under the curve (PR-AUC, also referred to as AUPRC), with best weights restored. ReduceLROnPlateau reduced the learning rate by a factor of 0.5 when validation PR-AUC plateaued, with a minimum learning rate of 1×10⁻⁶. These same model-selection criteria were applied consistently across all dataset regimes and fine-tuning strategies.

Class imbalance handling

To address class imbalance, inverse-frequency class weights were applied during optimization via the 'class_weight' option in training. Let N denote the total number of training samples in a given regime, let n_c denote the number of training samples belonging to class c, and let c ∈ {0,1} correspond to the 'NORMAL' and 'PNEUMONIA' classes, respectively. Class weights were computed as w_c = N / (2·n_c) and applied during training.

Data augmentation

A consistent augmentation pipeline is applied in all runs, including random horizontal flipping, small rotations (≈ ±7°), translations (≈ ±8%), zoom, contrast adjustment, and mild Gaussian noise.

Evaluation metrics 

Performance was evaluated on the fixed held-out test set using accuracy, area under the receiver operating characteristic curve (ROC-AUC), average precision (AP; PR-AUC/AUPRC), and test loss. Because pneumonia screening datasets can be imbalanced, ranking-based metrics were emphasized, particularly average precision, which is often more informative than ROC-AUC in imbalanced settings [14]. Accuracy was retained as a threshold-dependent complementary metric.

## Results

Test performance across dataset sizes and fine-tuning depths

Table 1 summarizes test-set performance for each dataset regime and fine-tuning strategy. Linear probing remains competitive across all regimes examined, particularly in low-data settings. As the dataset size increases, performance improves across all strategies. Relative to linear probing, fine-tuning provides modest improvements in ranking-based metrics, including area under the receiver operating characteristic curve and average precision. In the regimes examined, fine-tuning conv4-5 is often comparable to fine-tuning conv5, with small differences in these ranking-based metrics that become negligible at larger sizes, including the full-data regime.

**Table 1:** Test performance across dataset sizes and fine-tuning depths Train subset sizes are reported as batches × 32 (exact for reduced regimes) due to batch-level truncation. ROC-AUC - receiver operating characteristic curve; AP - average precision; PR-AUC - precision-recall area under the curve; LP - linear probing; FT - fine-tuning

Train subset (n)	Strategy	Test Loss	Accuracy	ROC-AUC	AP (PR-AUC)
128	LP (frozen)	0.567	0.742	0.765	0.815
128	FT conv5	0.574	0.718	0.773	0.824
128	FT conv4–5	0.565	0.736	0.777	0.827
256	LP (frozen)	0.506	0.771	0.837	0.883
256	FT conv5	0.498	0.777	0.843	0.888
256	FT conv4–5	0.486	0.793	0.847	0.891
512	LP (frozen)	0.443	0.782	0.892	0.928
512	FT conv5	0.416	0.811	0.897	0.932
512	FT conv4–5	0.41	0.812	0.901	0.935
1024	LP (frozen)	0.346	0.848	0.932	0.958
1024	FT conv5	0.356	0.833	0.933	0.958
1024	FT conv4–5	0.338	0.851	0.936	0.96
2080	LP (frozen)	0.288	0.894	0.95	0.969
2080	FT conv5	0.284	0.886	0.951	0.969
2080	FT conv4–5	0.286	0.873	0.952	0.97
4186 (Full)	LP (frozen)	0.316	0.854	0.96	0.974
4186 (Full)	FT conv5	0.248	0.909	0.962	0.976
4186 (Full)	FT conv4–5	0.248	0.909	0.962	0.976

From a practical standpoint, these results suggest that linear probing is a strong low-cost baseline in low-data regimes, where deeper unfreezing can yield only modest gains and may not improve accuracy at a fixed threshold. In higher-data regimes, fine-tuning tends to improve ranking-based metrics relative to linear probing, but the gap between fine-tuning conv5 and conv4-5 narrows, consistent with diminishing returns for additional unfreezing under this pipeline.

## Discussion

This study isolates how dataset size relates to transfer-learning performance and the incremental value of fine-tuning depth in a controlled chest X-ray classification task. Consistent with prior evidence that the optimal transfer-learning strategy can be task- and data-dependent [9], our results show strong learning-curve improvements with increasing dataset size across all strategies. However, the additional gains of partial fine-tuning over linear probing are modest in the regimes examined and do not increase monotonically with dataset size. Likewise, the difference between fine-tuning conv5 and conv4-5 is generally small and becomes negligible in the largest regime.

Dataset size as a determinant of fine-tuning benefit

A primary observation is that linear probing remains competitive in low-data regimes, suggesting that pretrained representations can be sufficiently expressive when data is scarce. Across the regimes examined, performance improves with increasing dataset size for all strategies. Relative to linear probing, partial fine-tuning provides small but consistent gains in ranking-based metrics (ROC-AUC and average precision), while accuracy is not consistently improved at a fixed threshold. However, deeper fine-tuning (conv4-5 vs. conv5) yields only incremental differences and converges to similar performance at larger sizes, indicating diminishing returns for additional unfreezing under this pipeline.

Metric considerations under class imbalance

Improvements in ROC-AUC and average precision are not always mirrored in accuracy at a fixed 0.5 threshold. This is expected because ROC-AUC and average precision reflect ranking quality across thresholds, while accuracy reflects a single operating point and can be influenced by class prevalence and calibration. In imbalanced screening contexts, ranking-based metrics are often more informative for cross-regime comparisons.

System-level implications

From an engineering perspective, these findings support a practical rule. Linear probing can be used as a strong, low-cost baseline in limited-data or constrained-compute scenarios. When moderate-to-larger data are available, and additional training cost is acceptable, shallow fine-tuning of conv5 is a reasonable choice because it tends to provide modest improvements in ranking-based metrics. Deeper unfreezing of conv4-5 may be considered when additional compute is available and incremental gains justify the added cost, noting the diminishing returns observed here and that accuracy gains are not guaranteed.

Limitations

This work has several limitations. First, only a single backbone architecture (ResNet-50) was evaluated. Second, the study focused on a single publicly available chest X-ray dataset and on a single binary classification task ('NORMAL' versus 'PNEUMONIA'), which may limit direct generalization to other datasets, label structures, or imaging tasks. Third, the main analysis was restricted to linear probing, FT conv5, and FT conv4-5, and full-network fine-tuning was not evaluated. Finally, results are based on a fixed split and a limited number of runs, without confidence intervals or formal statistical significance testing. Accordingly, the findings should be interpreted as controlled empirical guidance within the present experimental setting rather than as a universally optimal prescription for transfer-learning practice.

## Conclusions

This controlled empirical study evaluated how dataset size relates to the relative value of different fine-tuning depths in transfer learning for chest X-ray classification. Linear probing remained a competitive and computationally efficient approach, particularly in low-data regimes. As the dataset size increased, performance improved across all strategies, and partial fine-tuning provided modest and generally consistent gains in ranking-based metrics, including area under the receiver operating characteristic curve and average precision, relative to linear probing.

However, deeper fine-tuning (FT conv4-5) provided only incremental differences compared with shallow fine-tuning (FT conv5) in the regimes examined, suggesting diminishing returns for additional unfreezing under this experimental setup. Overall, the results suggest that the most appropriate fine-tuning depth depends on dataset size and deployment constraints in the studied setting, and they provide practical guidance for transfer-learning decisions in medical imaging pipelines.
